# Cost-Effectiveness of the Use of Gold Anchor™ Markers in Prostate Cancer

**DOI:** 10.7759/cureus.11229

**Published:** 2020-10-29

**Authors:** Martina Lundqvist, Lars-Åke Levin

**Affiliations:** 1 Department of Health, Medicine and Caring Sciences, Linköping University, Linköping, SWE

**Keywords:** cost-effectiveness, gold fiducial markers, markov model, health-economic evaluation, meta-analysis

## Abstract

Introduction

A common treatment for prostate cancer is external beam radiation therapy. A way to target the radiation is to use implantable gold fiducial markers (GFMs). The GFMs serve as reference points enabling tumor localization during treatment. Today, there are several GFMs available on the market but no clinical guidelines as to which one to use. The aim of this study was to estimate the cost-effectiveness of Gold Anchor GFMs (Naslund Medical AB, Huddinge, Sweden) implanted with a 22G needle, compared to other GFMs implanted with a 17-18G needle, in the prostate gland of patients with prostate cancer.

Methods

Costs, life years, and quality-adjusted life years (QALYs) were estimated over a lifelong time horizon for each treatment strategy using a decision-analytic model. Data used in the model were obtained from published literature or were estimated by an expert elicitation technique. The primary outcome measure was an incremental cost-effectiveness ratio (ICER).

Results

Gold Anchor GFM was found to be a dominant alternative with both lower costs [-8.7 US Dollars (USD)] and a gain in QALYs (0.015) when compared with other GFMs. The lower cost was achieved by fewer visits for imaging in treatment planning, and by reduced risk of infections and sepsis. The QALY gain was driven by a reduced risk of sepsis.

Conclusion

The use of Gold Anchor GFMs as reference points to target radiation is a cost-effective alternative when compared to other GFMs. However, this analysis is based on expert elicitation regarding some crucial parameters, and further clinical studies of the use of GFMs are needed.

## Introduction

Prostate cancer is the most common cancer diagnosis among men in Sweden. It accounts for 33% of all cancers, and its incidence has more than doubled since the 1980s [1]. It is therefore important from both a public health and economic perspective to optimize the care process of prostate cancer treatment.

External beam radiotherapy is one of several available treatment options for localized prostate cancer, which gives a dose-dependent result. The importance of correct positioning of the radiation has increased with the implementation of dose escalation since higher doses increase toxicity to surrounding organs [2]. One method to target the radiation is to use implantable gold fiducial markers (GFMs). The GFMs serve as reference points for positioning and enable tumor localization during treatment. It is considered to be a safe, well-tolerated, and reliable method to verify the position [3]. Nonetheless, complications such as bleeding, infections, and pain have been reported [3,4].

Today, there are several GFMs available in the market but no clinical guidelines exist for choosing a suitable marker [3]. Gold Anchor (Naslund Medical AB, Huddinge, Sweden) is a GFM with a unique design intended to reduce the risk of marker migration, and it is thin enough to be implanted in the prostate gland with a 22G syringe needle. The use of a thin needle has been shown to minimize the risk of implantation complications [5]. Another advantage with the Gold Anchor GFM is that less marker migration makes it possible to accomplish both the implantation and imaging in treatment planning on the same day [5]. 

Although the differences between Gold Anchor GFMs and other GFMs are relatively small, they should not be ignored. A series of small steps forward can, over time, add up to improvements that may result in great advantages that will make a difference in the end. It is therefore of value to not disregard or underestimate these differences [6].

To inform the decision maker’s choice between competing GFM alternatives, a health economic analysis has been undertaken. No other study evaluating the cost-effectiveness of GFMs has, to our knowledge, been published so far.

The aim of this study is to estimate the cost-effectiveness of Gold Anchor GFMs implanted with a 22G needle compared to other GFMs implanted with a 17-18G needle in the prostate gland of patients with prostate cancer.

## Materials and methods

Overview of analytical approach

The patient population of interest in this study was men with localized prostate cancer in need of radiation therapy. The treatment strategies investigated were the use of Gold Anchor GFMs implanted with a 22G needle compared to other GFMs implanted with a 17-18G needle to target the radiation therapy. A decision-analytic model was developed and used to determine costs and quality-adjusted life years (QALYs) for a lifelong time horizon. The primary outcome was the incremental cost-effectiveness ratio (ICER). It should be interpreted as the extra cost of obtaining an extra unit of effectiveness. If one treatment was more effective and less costly compared to the other, it was denoted as dominant. Costs and effects were discounted using a 3% annual rate. The costs were converted to US Dollars (USD) with an exchange rate of 1 USD = 8.65 Swedish krona (SEK) (2016 mean exchange rate). The analysis was performed from a societal perspective, including all costs, stemming from the treatment, which were incurred by society.

Decision-analytic model

**Figure 1:**
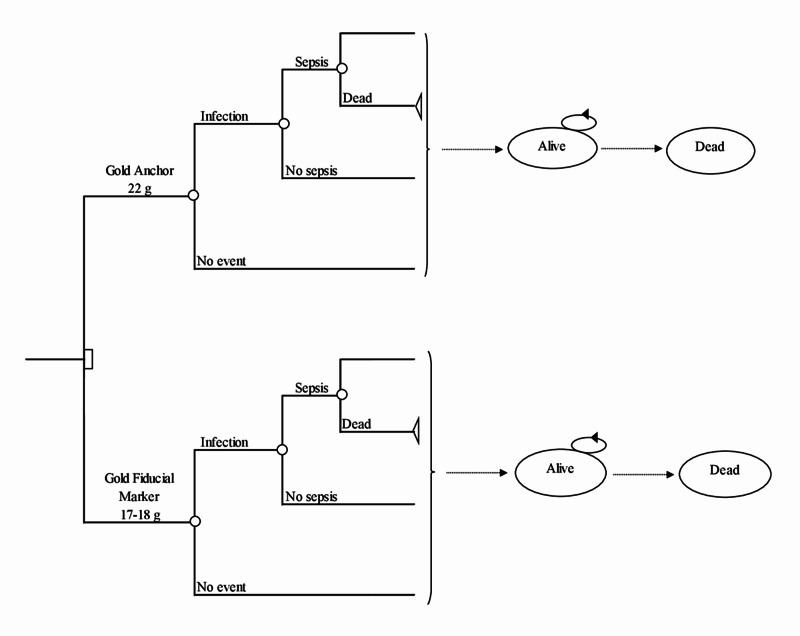
Model structure

In order to evaluate the cost-effectiveness of Gold Anchor GFMs compared with other GFMs, a dynamic Markov model was developed [7]. Initially, all patients were assumed to have undergone implantation of fiducial markers. After implantation, two initial outcomes were possible: ‘no event’ or ‘Infection’; ‘no event’ implied that the patient had no complications that required treatment. The ‘Infection’ outcome was associated with a probability of sepsis. Survival after sepsis was determined by the probability of death due to sepsis. Patients who stayed in the ‘Infection’ outcome were assumed to have been treated with antibiotics. Since all possible events caused by the implementation occurred immediately after the implantation, the only possible health states during the remaining years (normally referred to as Markov cycles) in the lifelong model were ‘Alive’ and ‘Dead’ (Figure 1). The model was repeated until all the patients had died. Each health state in the model was associated with a cost and a health outcome.

Data

Risks

**Figure 2:**
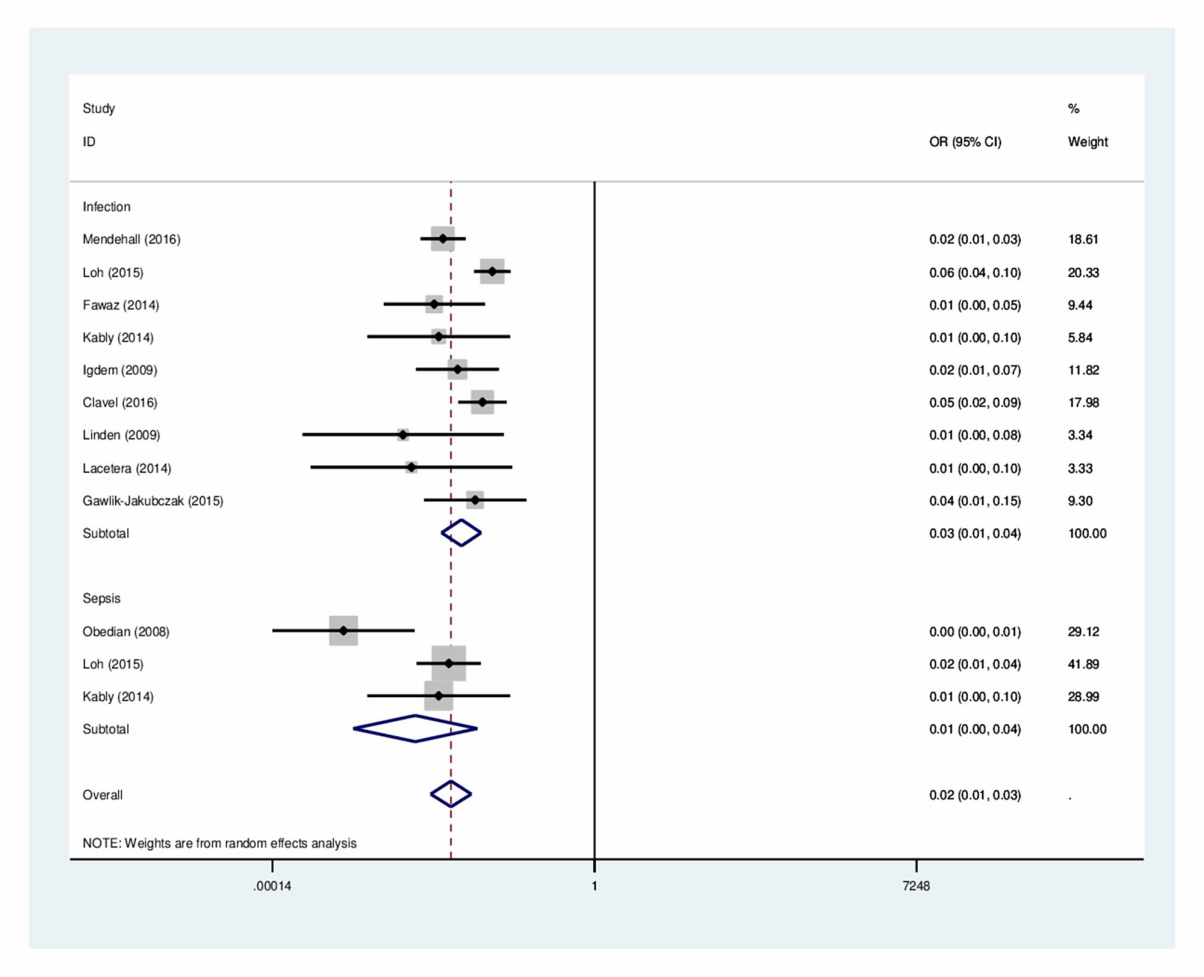
Results of meta-analysis to determine the risk of infection and the risk of sepsis

The model used age-based standard mortality rates for men in Sweden in 2014 [8]. An additional annual risk of dying because of prostate cancer was added to the standard mortality. Mortality due to sepsis was modeled using data from a study by Rodríguez et al. [9], which was defined as a 28-day mortality rate. The risk of infections and risk of sepsis when implanting Gold Anchor GFMs were obtained from a study conducted by Castellanos et al. [5]. To determine risks of infections and sepsis when implanting GFMs with a 17-18G needle, we combined estimated risks from published sources in a meta-analysis. Risks from individual studies were combined using a random effect model following the method of DerSimonian and Laird [10], with the estimate of heterogeneity taken from the Mantel-Haenszel model (Figure 2). Regarding infections, an odds ratio of 0.03 was estimated, based on nine studies [4,11-18]. The corresponding estimate for sepsis was 0.01, based on three studies [4,12,19]. As the model requires transition probabilities as input rather than the estimated odds, the estimated odds from the meta-analyses were converted to probabilities using the formula odds/(1+odds). For details about the meta-analysis, see Appendix 1.

Other risks used in the model were obtained from published literature or were estimated by an expert elicitation technique. The expert opinions were obtained with questionnaires devised by the research group. The respondents were identified with help from the Regional Cancer Center South East (RCC). Two different questionnaires were devised as the questions were addressed to respondents with different professions, both oncologists and urologists. Risks used in the model are presented in Table 1.

Utility Weights

Age-adjusted Swedish population norms for men of EQ-5D index scores were used as QALY weights in the model [20]. A QALY-weight decrement due to prostate cancer of 0.1, assumed to remain for life, was used [21]. Additionally, a QALY-weight decrement of 0.1 during the possible waiting time between implantation and imaging in treatment planning was used. The decrements were linked to the number of days a patient spent in a specific state. QALY weights for sepsis were obtained from a study conducted by Drabinski et al. [22]. The QALY weights and the decrements used in the model are presented in Table 1.

Costs and Resource Usage

The drug costs were gathered from FASS (pharmaceutical specialties in Sweden; www.fass.se) [23]. Costs for administrative work and costs for sick leave were obtained from Statistics Sweden (SCB) [24]. Travel costs were obtained from the Swedish Tax Agency [25]. The cost of sepsis was based on information from the National Board of Health and Welfare [26]. The remaining unit costs were obtained from the cost per patient (KPP) database in the county council of Östergötland, Sweden [27], and from a regional price list (pricing and payment for healthcare in the Southeast region of Sweden, 2014) [28].

Resource usage in the model was obtained from published literature or estimated through expert opinions as described previously. Unit costs and resource usage are presented in Table 1.

**Table 1:** Main parameters in the model ^a^Risk of infection requiring antibiotic treatment

Parameter	Estimate	Reference
Resource usage		
Gold fiducial markers used, 22G	3	Expert opinion
Gold fiducial markers used, 17-18G	3.3	Expert opinion
Visits for implantation	1	
Visits to the doctor due to infection	1.5	Expert opinion
Days of hospitalization due to sepsis	12	[29]
Days between implantation and imaging in treatment planning	6	Expert opinion
Days of sick leave due to imaging for treatment planning	1	
Distance covered for the hospital visit, round-trip (km)	130	Unpublished data from Statistics Sweden
Required time to rebook a visit (hours)	0.71	Personal communication, Urology Clinic at Linköping University Hospital
Probabilities		
Rebook of visit for imaging in treatment planning	0.015	Personal communication, Urology Clinic at Linköping University Hospital
Gold Anchor 22G		
Analgesics	0.4	Expert opinion
Without/no severe complication	0.997^a^	
Infection requiring antibiotic treatment	0.003	[5]
Infection leading to sepsis	0	[5]
Death due to sepsis	0.186	[9]
17-18G		
Analgesics	0.73	Expert opinion
Without/no severe complication	0.968^a^	
Infection requiring antibiotic treatment	0.024	Meta-analysis [4,11-18]
Infection leading to sepsis	0.285	Meta-analysis [4,12,19]
Death due to sepsis	0.1863	[9]
Costs (USD)		
Gold fiducial marker – Gold Anchor 22G (per marker)	Censored	Personal communication, Näslund Medical AB
Gold fiducial marker – 17-18G (per marker)	28	[27]
Implantation of gold fiducial marker	493	[27]
Antibiotic prophylaxis	2	Expert opinion
Analgesics	22	Expert opinion
Antibiotic treatment for an infection	15	Expert opinion
Treatment for sepsis	6 276	[26]
Administrative work, cost per hour	25	[24]
Sick leave, cost per day	250	[30]
Travel cost, 10 km	2	[25]
Quality-adjusted life year weights		
50-59 years	0.845	[20]
60-69 years	0.829	[20]
70-79 years	0.797	[20]
Quality-adjusted life year weight decrement; prostate cancer	0.1	[21]
Sepsis; during hospitalization	0.53	[22]
Sepsis; t=1	0.62	[22]
Quality-adjusted life year weight decrement; anxiety due to waiting time	0.1	Assumption

Analysis

The model was run for fictive patients with the starting age of 65 years. In the base-case analysis, we assumed that it is feasible to perform implantation of Gold Anchor GFMs and imaging in treatment planning on the same day, based on data from a study conducted by Castellanos et al. [5].

To analyze how different assumptions, simplifications, and certain parameters affected the results, one-way sensitivity analyses were performed where key assumptions varied, e.g., the time horizon, the starting age, the discount rates, the risk of sepsis, and the possibility or otherwise of carrying out the implantation of Gold Anchor GFMs and conducting imaging in treatment planning on the same day. Since data were indeterminate, we also conducted a sensitivity analysis where we assumed the risk of infections and sepsis to be equal for both options. The results were only analyzed deterministically since the model did not include any probabilistic values. The model was programmed and analyzed in Microsoft Excel (Microsoft Corporation, Redmond, WA).

## Results

Cost-effectiveness

The result of the base-case analysis, as presented in Table 2, showed that Gold Anchor GFMs implanted with a needle sized 22G was dominant when compared to other GFMs implanted with a needle sized 17-18G, with lower costs (-8.7 USD) and a gain in QALYs (0.015). The lower costs were mainly achieved by the avoidance of an additional visit for imaging in treatment planning and the reduced risk of infections and sepsis. The QALY gain was driven by the reduced risk of sepsis.

**Table 2:** Base-case results: cost-effectiveness of Gold Anchor gold fiducial markers compared to other gold fiducial markers

	Costs (USD)	∆Cost (USD)	Quality-adjusted life years	∆Quality-adjusted life years	Life years	∆Life years	Cost per life year gained	Cost per quality-adjusted life year gained
Gold Anchor, 22G	679	-8.7	9.287	0.015	13.477	0.017	Dominant	Dominant
Other gold fiducial markers, 17-18G	688		9.273	-	13.459	-		

Sensitivity analysis

Sensitivity analysis with different discount rates and starting ages, as reported in Table 3, also showed no differences when compared to the base-case analysis. In scenarios 1-4, Gold Anchor GFMs were dominant when compared to other GFMs. In scenario 5, assuming no differences regarding the risk of sepsis, Gold Anchor GFMs were still dominant when compared to other GFMs. When assuming no difference regarding both risk of infection and risk of sepsis (scenario 6), Gold Anchor GFMs were associated with an incremental cost of 43.8 USD and a gain of 0.002 QALYs, yielding a cost per QALY of approximately 27 012 USD for Gold Anchor GFMs compared to other GFMs. In scenario 7, assuming that it was not possible to carry out the implantation of the Gold Anchor GFMs and imaging in treatment planning on the same day, Gold Anchor GFMs yielded an additional cost of 19.9 USD and a QALY gain of 0.013, resulting in a cost per QALY of approximately 1 503 USD. Finally, in scenario 8, using a one-year time horizon, Gold Anchor GFMs were dominant when compared to other GFMs.

**Table 3:** Results of sensitivity analysis

Scenario		Incremental cost (USD)	Incremental quality-adjusted life year	Incremental cost-effectiveness ratio
1	Discount rate 0%	-8.7	0.019	Dominant
2	Discount rate 5%	-8.7	0.013	Dominant
3	Starting age 60 years	-279.5	0.017	Dominant
4	Starting age 70 years	-8.7	0.013	Dominant
5	No sepsis difference	-3.5	0.013	Dominant
6	No infection and sepsis difference	43.8	0.002	27 012
7	Gold Anchor implantation ≠ treatment planning	19.9	0.013	1 503
8	One-year time horizon	-8.7	0.004	Dominant

## Discussion

Today, there are several GFMs available in the market but no clinical guidelines as to which one to use. To inform the decision maker’s choice between competing GFM alternatives, we assessed the cost-effectiveness of the Gold Anchor GFMs implanted with a 22G needle compared to other GFMs implanted with a 17-18G. The analysis showed that Gold Anchor GFMs reduces the cost per patient compared to other GFMs, but the differences are small. The use of Gold Anchor GFMs also results in a QALY gain due to the reduced risk of sepsis. Taken together, the base-case analysis shows that Gold Anchor GFMs are dominant when compared to other GFMs, which implies that the Gold Anchor GFMs are both more efficient and less costly than other GFMs.

To our knowledge, this is the first study estimating the cost-effectiveness of different GFMs. One strength of the study is the long-term extrapolation that makes it possible to account for all costs and effects of the different strategies included in the analysis. A study limitation is that the data used in the analysis were gathered from separate trials. This implies that the underlying method in this analysis is an indirect comparison of single-arm trials, known as naïve comparisons. Naïve comparisons have several limitations that should be kept in mind when interpreting the results. The major limitation with the approach is that it does not allow adjustment for cross-trial differences, which can lead to confounding bias. To obtain more reliable estimates, we performed a meta-analysis. The aggregation of information leads to a higher statistical power and more robust point estimates than is possible from measures derived from an individual study. A related limitation though concerns selection bias regarding which studies are included in the meta-analysis. However, a genuine attempt was made to locate reliable studies estimating complications caused by the implantation of GFMs. In the study, we also used expert opinions to estimate unknown values. Using expert opinions as data sources may imply a methodological weakness, but it was considered to be the best available alternative. In view of the discussion above, the study should be considered as a pilot study.

Since the model was based on uncertain parameters, we performed one-way sensitivity analyses where different assumptions, simplifications, and variation of uncertain parameters varied. Different discount rates and different starting ages did not affect the results. If assuming no difference regarding the risk of infections and sepsis between the options, or delay between implantation and imaging in treatment planning, Gold Anchor GFMs yielded a cost per QALY of approximately 27 000 USD and 1 500 USD, respectively. One still has to consider whether the cost per QALY should be considered reasonable. In Sweden, no explicit threshold value for a QALY is available, though a figure often mentioned is 60 000 USD. However, aspects other than cost-effectiveness need to be considered when allocating healthcare resources, for example, the severity of the condition and implications for the overall healthcare budget. Furthermore, since the data used in this model were indeterminate and several assumptions had to be made, the results should be interpreted with caution. Hence, in order to find more reliable data, the use of different GFMs needs to be studied further.

Since no other studies estimating the cost-effectiveness of different GFMs has been conducted, it was not possible to validate the results from our study with respect to previous research. This, in combination with the methodical weaknesses, implies that additional research is needed. Future analyses should, if possible, be based on more rigorous data, preferably from clinical studies comparing the use of different GFMs. Such studies should focus on the incidence of complications. In connection with a clinical study, it is also essential to establish the use of prophylaxis and analgesics for different GFMs. Further, in a study estimating QALY-weights for patients suffering from prostatic cancer, factoring in procedure-related anxiety would contribute to the reliability of the study.

This study demonstrates the feasibility of conducting an early analysis to evaluate the costs and effects of new technologies. It illustrates that considering even small differences can be of value and should therefore not be ignored. Small improvements can contribute to more effective healthcare and, even if medical devices are moving targets for evaluation, a series of small steps forward may add up to improvements that will result in great advantages over time. In the long run, it is also possible that small improvements will provide significant returns and should therefore not be ignored.

## Conclusions

This study shows that Gold Anchor GFM can be a dominant alternative to other GFMs. The costs per QALY gained in the sensitivity analyses are also considered cost-effective, according to accepted practice in Sweden and other European countries.

## Figures and Tables

**Figure 3:**
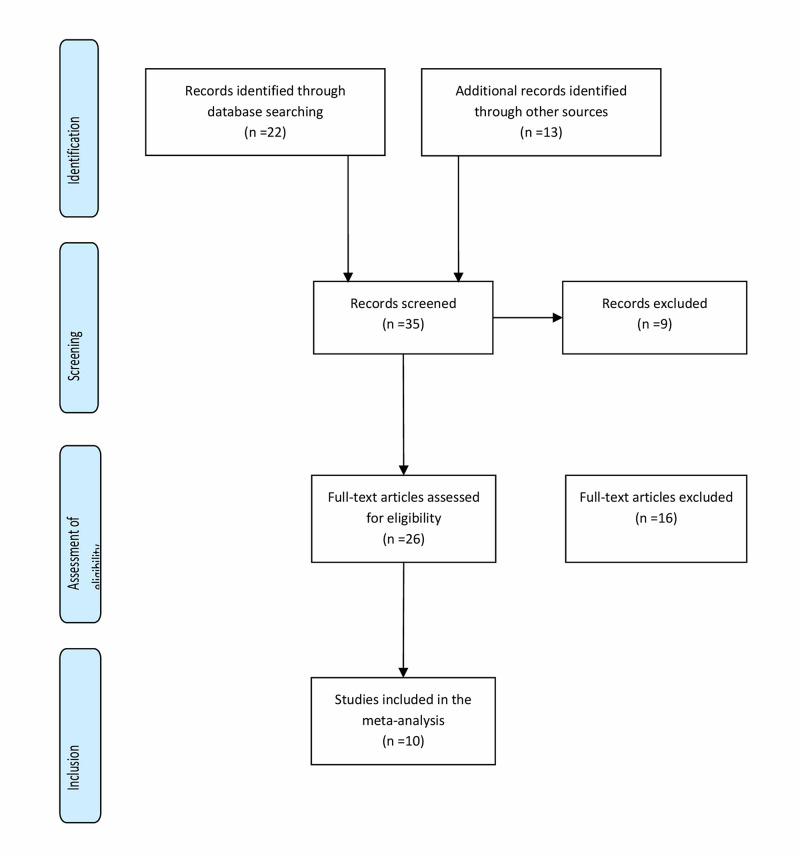
Flow chart of the study selection: PRISMA 2009 flow diagram PRISMA: Preferred Reporting Items for Systematic Reviews and Meta-Analyses

## Appendices

Evidence synthesis

To establish the risk of infections and risk of sepsis when implanting GFMs with a 17-18G needle, we searched the electronic database PubMed. Search terms included combinations of “Gold Fiducial Marker”, “Complications”, “Infection”, “Sepsis” and “Prostate cancer”. In addition to standard database searches, reference lists were searched manually. Inclusion criteria applied were:

· Implantation of markers with transrectal ultrasound

· Implanting GFMs with a 17-18G needle

· Implantation of three or four markers

· Patients receiving antibiotic prophylaxis but not combinations of antibiotic prophylaxis

· Data based on medical records

· Only empirical treatment (e.g., urinary infection documented by urinary culture)

Study selection

The search of PubMed provided 22 unique articles. Additionally, 13 articles were identified when reference lists were searched manually. Based on titles and abstracts, nine articles did not meet eligibility criteria and were therefore excluded. Another 16 articles were excluded after reading the full text. Finally, 10 different studies met the inclusion criteria. The study selection process is illustrated in the flow chart in Figure 3.

Meta-analysis

The outcomes of interest were non-comparative binary outcomes, i.e. probability of infection and probability of sepsis. Therefore, a synthesis of odds was carried out on the log scale. To determine the risk of infections and the risk of sepsis when implanting GFMs with a 17-18G needle, a meta-analysis, using a random-effect method, was conducted [10]. The random-effect model was considered suitable since the heterogeneity chi-squared test (standard test for heterogeneity) indicated heterogeneity between the studies. The forest plot also indicated that there was heterogeneity between the trials, based on the variability between the estimates on the plot (Figure 2). To handle computational problems concerning studies with zero-cell counts, the zero counts were adjusted by adding a fixed value of 0.5. As the model requires transition probabilities as input rather than the estimated odds, the estimated odds from the meta-analyses were converted into probabilities using the formula odds/(1+odds).

## References

[1] (2017). The National Board of Health and Welfare. Cancer incidence in Sweden 2014 - new diagnosed cancer cases in 2014 (content in Swedish). Sveriges officiella statistik. https://www.socialstyrelsen.se/globalassets/sharepoint-dokument/artikelkatalog/statistik/2015-12-26.pdf.

[2] Ng M, Brown E, Williams A, Chao M, Lawrentschuk N, Chee R (2014). Fiducial markers and spacers in prostate radiotherapy: current applications. BJU Int.

[3] Langenhuijsen JF, van Lin EN, Kiemeney LA, van der Vight LP, McColl GM, Visser AG, Witjes JA (2007). Ultrasound-guided transrectal implantation of gold markers for prostate localization during external beam radiotherapy: complication rate and risk factors. Int J Radiat Oncol Biol Phys.

[4] Loh J, Baker K, Sridharan S (2015). Infections after fiducial marker implantation for prostate radiotherapy: are we underestimating the risks?. Radiat Oncol.

[5] Castellanos E, Wersäll P, Tilikidis A, Andersson AH (2018). Low infection rate after transrectal implantation of Gold Anchor™ fiducial markers in prostate cancer patients after non-broad-spectrum antibiotic prophylaxis. Cureus.

[6] Wertheimer A, Levy R, O'Connor T (2001). Too many drugs? The clinical and economic value of incremental innovations. Res Hum Capital Dev.

[7] Sonnenberg FA, Beck JR (1993). Markov models in medical decision making: a practical guide. Med Decis Making.

[8] (2017). Swedish Statistics (SCB). Lifetime table 2010-2014, divided into men and women. http://webcache.googleusercontent.com/search?q=cache:bfeK03iqj3oJ:www.scb.se/Statistik/BE/BE0101/2014A01G/Be0101Livslangdstabeller-14.xlsx+&cd=1&hl=en&ct=clnk&gl=se.

[9] Rodríguez F, Barrera L, De La Rosa G (2011). The epidemiology of sepsis in Colombia: a prospective multicenter cohort study in ten university hospitals. Crit Care Med.

[10] Sutton AJ, Abrams KR, Jones DR, Sheldon TA, Song F (2000). Methods for Meta-Analysis in Medical Research. Methods for Meta-Analysis in Medical Research..

[11] Fawaz ZS, Yassa M, Nguyen DH, Vavassis P (2014). Fiducial marker implantation in prostate radiation therapy: complication rates and technique. Cancer Radiother.

[12] Kably I, Bordegaray M, Shah K, Salsamendi J, Narayanan G (2014). Single-center experience in prostate fiducial marker placement: technique and midterm follow-up. J Vasc Interv Radiol.

[13] Igdem S, Akpinar H, Alço G, Agaçayak F, Turkan S, Okkan S (2009). Implantation of fiducial markers for image guidance in prostate radiotherapy: patient-reported toxicity. Br J Radiol.

[14] Linden RA, Weiner PR, Gomella LG, Dicker AP, Suh DB, Trabulsi EJ, Valicenti RK (2009). Technique of outpatient placement of intraprostatic fiducial markers before external beam radiotherapy. Urology.

[15] Lacetera V, Cardinali M, Mantello G (2014). Prostatic fiducial markers implantation by transrectal ultrasound for adaptive image guided radiotherapy in localized cancer: 7-years experience. Arch Ital Urol Androl.

[16] Mendenhall WM, Glassman G, Morris CG (2016). Bacterial urinary tract infection after transrectal placement of fiducial markers prior to proton radiotherapy for prostate cancer. Int J Part Ther.

[17] Clavel S, Gauthier-Pare AS, Duplan D, Fanizzi J, Rivest N, Haeck O (2016). Infections after fiducial markers implantation for prostate radiation therapy: optimizing the antimicrobial prophylaxis. Int J Radiat Oncol Biol Phys.

[18] Gawlik-Jakubczak T, Matuszewski M (2015). Fiducial markers in the treatment of prostate cancer: technique and short term observation. Oncol Clin Pract.

[19] Obedian E, Kapoor DA, Olsson CA, Schumer M, Lieberman E, Zimberg SH (2008). The risk of urosepsis in patients undergoing fiducial marker placement for image guided radiation therapy (IGRT) of prostate cancer. Int J Radiat Oncol Biol Phys.

[20] Burström K, Johannesson M, Diderichsen F (2006). A comparison of individual and social time trade-off values for health states in the general population. Health Policy.

[21] Pataky R, Gulati R, Etzioni R (2014). Is prostate cancer screening cost-effective? A microsimulation model of prostate-specific antigen-based screening for British Columbia, Canada. Int J Cancer.

[22] Drabinski A, Williams G, Formica C (2008). Observational evaluation of health state utilities among a cohort of sepsis patients. Value Health.

[23] FASS FASS (2016). FASS. Bactrim forte and ciprofloxacin. http://www.fass.se.

[24] (2016). Swedish Statistics (SCB). Salary database - average salary for office assistant and secretary, county council. 2014 [Dataset] and labor cost per hour in Europe 2012. Europe.

[25] (2016). The Swedish Tax Agency. Deductions for trips to work. https://www.skatteverket.se/privat/skatter/biltrafik/avdragforresortillochfranarbetet.4.3810a01c150939e893f25603.html.

[26] (2016). The National Board of Health and Welfare. NordDRG Slutenvård (content In Swedish). http://www.socialstyrelsen.se/klassificeringochkoder/norddrg/vikter.

[27] (2020). Swedish Association of Local Authorities and Regions (SALAR). KPP-database (cost per patient) (content In Swedish). https://skl.se/ekonomijuridikstatistik/statistik/kostnadperpatientkpp/kppdatabas.1079.html.

[28] (2017). Southeastern healthcare region. Price list for the southeastern healthcare region 2014 (content In Swedish). http://plus.lj.se/infopage.jsf.

[29] Husak L, Marcuzzi A, Herring J, Wen E, Yin L, Capan DD, Cernat G (2010). National analysis of sepsis hospitalizations and factors contributing to sepsis in-hospital mortality in Canada. Healthc Q.

[30] (2016). Swedish Statistics (SCB). Actual average working hours per week in main occupation for employed 15-74 years, hours after domestic/foreign born, gender, age and year. http://www.statistikdatabasen.scb.se/pxweb/sv/ssd/START__AM__AM0401__AM0401R/NAKUMedelvInrUtrJmAr/table/tableViewLayout1/?rxid=2619ad66-10a0-440b-889a-41485ee855d9..

